# Special Issue: Therapeutic Potential for Cannabis and Cannabinoids 2.0

**DOI:** 10.3390/biomedicines13123056

**Published:** 2025-12-11

**Authors:** Wesley M. Raup-Konsavage

**Affiliations:** Department of Neuroscience & Experimental Therapeutics, Penn State College of Medicine, Hershey, PA 17033, USA; wkonsavage@pennstatehealth.psu.edu; Tel.: +1-717-531-4172

While *Cannabis sativa* L. has a long history of medicinal use across cultures, its acceptance in Western medicine diminished in the early 20th century [1,2,3,4,5,6,7]. In contrast, the 21st century has seen a dramatic resurgence of interest, not only in the plant itself, but also in its diverse array of secondary metabolites, including terpenes, flavonoids, and most notably phytocannabinoids. Since California became the first U.S. state to legalize medicinal cannabis in 1996, a growing number of states and countries have followed suit, expanding access to both medicinal and adult recreational cannabis [1,8,9]. However, the pace of scientific research has lagged behind public enthusiasm and policy reform.

A review of PubMed records reveals that between 1980 and 2000, only 68 to 207 articles per year were published using the search term “cannabis”, averaging just 114 publications annually (Figure 1). A significant rise began around 2004, culminating in 3944 articles published in 2022. Although there was a slight decline in 2023 (3718) and 2024 (3795), the trend still reflects a major increase in scientific attention. Despite this growth, much of the research remains focused on just two compounds: cannabidiol (CBD) and Δ^9^-tetrahydrocannabinol (THC). In 2022 alone, 1091 publications addressed CBD and 893 focused on THC, representing 28.7% and 22.6% of all “cannabis” (original search term) articles. In contrast, other cannabinoids such as cannabigerol (CBG), cannabichromene (CBC), and cannabinol (CBN) accounted for 1.4%, 0.7%, and 1.7% of the literature, respectively. This imbalance underscores the urgent need to explore the broader pharmacological landscape of *Cannabis sativa* and its many bioactive constituents, including over 150 identified phytocannabinoids [10,11,12,13].

1. **Original Research Articles**

This Special Issue of *Biomedicines* presents ten original research articles that explore diverse facets of cannabinoid pharmacology, including studies on novel receptor agonists, under-investigated minor cannabinoids (e.g., CBC, CBN, CBG) and the therapeutic applications of CBD.

Two studies examined novel cannabinoid receptor ligands. Masocha et al. demonstrated that licofelone, a dual cycloxygernase/lipoxygenase inhibitor, binds both CB1 and CB2 receptors in silico and attenuates paclitaxel-induced neuropathic pain in rats through cannabinoid receptor-dependent mechanisms (**Contribution 1**). Guilherme et al. investigated oleoyl serotonin, a compound isolated from *Stephanoconus* snail venom. Structurally similar to endocannabinoids, this molecule competitively inhibited CB1 and non-competitively blocked CB2, and was able to reverse WIN55,212-induced memory deficits in mice (**Contribution 5**).

Several studies focused on minor cannabinoids. Trainito et al. found that CBN was non-toxic to differentiated NSC-34 neuronal cells and modulated genes involved in cell cycle regulation, suggesting potential neuroprotective effects (**Contribution 3**). Artimagnella et al. studied cannabinerol (CBNR), a CBG analogue derived from neryl pyrophosphate the cis-isomer of geranl pyrophosphate and observed upregulation of genes related to synaptic organization in NSC-34 cells (**Contribution 7**). Raup-Konsavage et al. reported that CBC exerted analgesic effects in multiple pain models and, through AI predictive modeling, was predicted to have a unique receptor profile compared to other phytocannabinoids. Therefore, CBC may offer not only novel therapeutic benefit as an analgesic but may work in synergy with other minor cannabinoids to target multiple receptors involved in pain (**Contribution 8**).

In another study assessing the antinociceptive activities of cannabinoids, Hayduk et al. examined CBD and CBG in oxaliplatin-induced neuropathy. While both cannabinoids showed efficacy, their effects differed in magnitude and dose-dependency. Interestingly, the combination exhibited both sub-additive and synergistic interactions depending on dose. This study also showed that both compounds reduced opioid withdrawal symptoms in mice (**Contribution 4**).

In a rat model of diabetic neuropathy, Khan et al. showed that CBD and the terpene β-caryophyllene reduced hyperalgesia and allodynia through an increase in antioxidant and anti-inflammatory activity (**Contribution 2**). Martínez-Aguirre et al. demonstrated that low doses of CBD suppressed excessive glutamate release in tissue samples from patients with drug-resistant epilepsy and similarly reduced hippocampal glutamate release in rats with spontaneous seizures. Additionally, the authors found that that some isolated synapses from patients were resistant to CBD treatment, but this did not appear to be associated with the type of epilepsy nor other clinical factors of the patients (**Contribution 9**).

Wong-Salgado et al. characterized cannabinoid and terpene profiles of cannabis inflorescences from four Peruvian regions, finding marked variability that could influence therapeutic use (**Contribution 6**). Finally, Abuhasira et al. assessed medical cannabis use in older adults (mean age 79.3). While cannabis use did not impair daily functioning, 36.1% of participants reported adverse events with dizziness being the most common; this raises concerns about fall risk (**Contribution 10**).

2. **Review Articles**

Four review articles provide context and synthesize current knowledge in key areas. Safi et al. reviewed the evidence supporting the use of THC and CBD for pain management, emphasizing the need for further mechanistic and clinical investigation (**Contribution 11**). Leinen et al. provided a broader overview of cannabinoid therapeutics across diseases such as inflammatory bowel disease, HIV, SARS-CoV-2, and cancer, while also highlighting concerns like cannabis use disorder (CUD) (**Contribution 13**). Rathod et al. explored the role of the endocannabinoid system in neuroinflammation, focusing on CB1 and CB2 signaling pathways (**Contribution 12**). Christensen et al. critically examined the concept of the “entourage effect,” proposing that pharmacological terms like synergy, bioenhancement, and pharmacokinetic/pharmacodynamic modulation better described the observed effects than the ill-defined “entourage effect”. The authors noted the anecdotal nature and inconsistency of the existing literature on this topic (**Contribution 14**).

3. **Closing Remarks**

The articles in this Special Issue reflect the evolving science of cannabis and cannabinoids as therapeutic agents, highlighting both emerging potential and persistent gaps in knowledge. From novel ligands to minor cannabinoids and synergistic interactions, this Special Issue expands the scientific foundation for cannabis research. However, critical challenges remain, including the lack of rigorous clinical trials, standardization of dosing and delivery, deeper exploration of cannabinoid-terpene interactions, and the pharmacology of lesser-studied cannabinoids. Meeting these challenges is essential to responsibly advancing cannabis-based therapeutics in modern medicine.

## Figures and Tables

**Figure 1:**
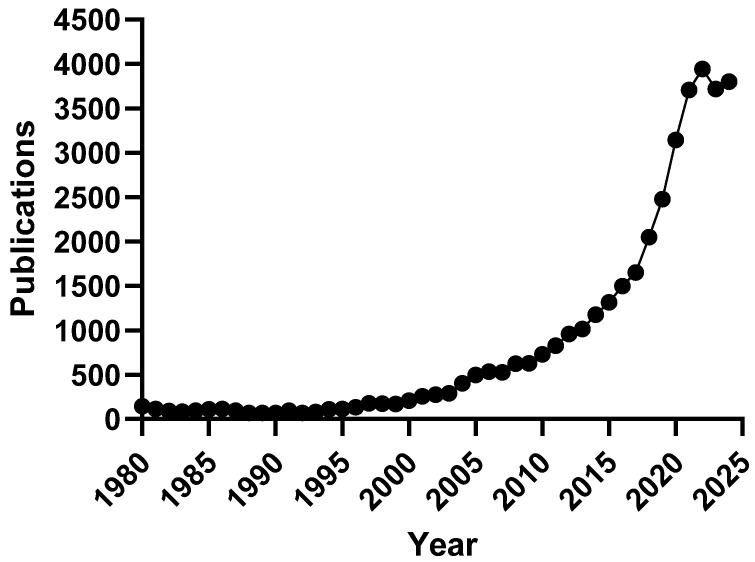
**Cannabis Publications Indexed on PubMed**. The number of publications on PubMed (pubmed.ncbi.nlm.nih.gov) for the search term “cannabis” between 1980 and 2024 is shown. It should be noted that in 1996, California became the first jurisdiction to legalize cannabis for medicinal purposes, and that the first noticeable increase in academic publications occurred about 10 years later [9].

## References

[1] Bostwick J.M. (2012). Blurred boundaries: The therapeutics and politics of medical marijuana. Mayo Clin. Proc..

[2] Abi-Jaoude E., Chen L., Cheung P., Bhikram T., Sandor P. (2017). Preliminary Evidence on Cannabis Effectiveness and Tolerability for Adults With Tourette Syndrome. J. Neuropsychiatry Clin. Neurosci..

[3] Hill K.P., Palastro M.D., Johnson B., Ditre J.W. (2017). Cannabis and Pain: A Clinical Review. Cannabis Cannabinoid Res..

[4] Pisanti S., Bifulco M. (2017). Modern History of Medical Cannabis: From Widespread Use to Prohibitionism and Back. Trends Pharmacol. Sci..

[5] Baron E.P. (2018). Medicinal Properties of Cannabinoids, Terpenes, and Flavonoids in Cannabis, and Benefits in Migraine, Headache, and Pain: An Update on Current Evidence and Cannabis Science. Headache.

[6] Fraguas-Sánchez A.I., Torres-Suárez A.I. (2018). Medical Use of Cannabinoids. Drugs.

[7] Legare C.A., Raup-Konsavage W.M., Vrana K.E. (2022). Therapeutic Potential of Cannabis, Cannabidiol, and Cannabinoid-Based Pharmaceuticals. Pharmacology.

[8] Carliner H., Brown Q.L., Sarvet A.L., Hasin D.S. (2017). Cannabis use, attitudes, and legal status in the U.S.: A review. Prev. Med..

[9] Orenstein D.G., Glantz S.A. (2020). Cannabis Legalization in State Legislatures: Public Health Opportunity and Risk. Marquette Law. Rev..

[10] ElSohly M.A., Radwan M.M., Gul W., Chandra S., Galal A. (2017). Phytochemistry of *Cannabis sativa* L.. Prog. Chem. Org. Nat. Prod..

[11] Gülck T., Møller B.L. (2020). Phytocannabinoids: Origins and Biosynthesis. Trends Plant Sci..

[12] Alsherbiny M.A., Bhuyan D.J., Low M.N., Chang D., Li C.G. (2021). Synergistic Interactions of Cannabidiol with Chemotherapeutic Drugs in MCF7 Cells: Mode of Interaction and Proteomics Analysis of Mechanisms. Int. J. Mol. Sci..

[13] Rock E.M., Parker L.A. (2021). Constituents of Cannabis Sativa. Adv. Exp. Med. Biol..

